# What you have, not who you know: food-enhanced social capital and changes in social behavioural relationships in a non-human primate

**DOI:** 10.1098/rsos.231460

**Published:** 2024-01-17

**Authors:** Rosemary Blersch, Jessica J. Vandeleest, Amy C. Nathman, Márton Pósfai, Raissa D'Souza, Brenda McCowan, Brianne A. Beisner

**Affiliations:** ^1^ Neuroscience and Behavior Unit, California National Primate Research Center, Davis, CA, USA; ^2^ Dept. of Network and Data Science, Central European University, Budapest, Nadoru 13104, Hungary; ^3^ University of California Davis, Davis, CA, USA; ^4^ The Sante Fe Institute, 1399 Hyde Park Rd, Santa Fe, NM 87501, USA; ^5^ Department of Population Health & Reproduction, School of Veterinary Medicine, University of California Davis, Davis, CA, USA; ^6^ Emory National Primate Research Center, Emory University, 2409 Taylor Rd, Suwanee, GA 30024, USA

**Keywords:** non-human primates, social networks, social capital, aggression, silent bared-teeth display

## Abstract

Social network position in non-human primates has far-reaching fitness consequences. Critically, social networks are both heterogeneous and dynamic, meaning an individual's current network position is likely to change due to both intrinsic and extrinsic factors. However, our understanding of the drivers of changes in social network position is largely confined to opportunistic studies. Experimental research on the consequences of *in situ,* controlled network perturbations is limited. Here we conducted a food-based experiment in rhesus macaques to assess whether allowing an individual the ability to provide high-quality food to her group changed her social behavioural relationships. We considered both her social network position across five behavioural networks, as well as her dominance and kin interactions. We found that gaining control over a preferential food resource had far-reaching social consequences. There was an increase in both submission and aggression centrality and changes in the socio-demographic characteristics of her agonistic interaction partners. Further, we found that her grooming balance shifted in her favour as she received more grooming than she gave. Together, these results provide a novel, preliminary insight into how *in situ*, experimental manipulations can modify social network position and point to broader network-level shifts in both social capital and social power.

## 1. Introduction

Social network analysis, as a means to quantify social structure and position in gregarious mammals, has increased exponentially and enables us to move beyond the more traditional dyadic approach [1,2] to consider how relationships scale to a broader social structure and the processes that drive them. Given the fitness consequences that social relationships and interactions have in non-human primates (see [3–5]), as well as their influence on disease and information transmission [6–9], understanding how primates as individuals function within families, communities and groups is critical. Networks are fundamentally heterogeneous and individuals vary in the social roles they play in a social network, both in terms of how central or peripheral each member is as well as how critical their presence is to the maintenance of social structure [10,11]. This, in combination with their position in the dominance hierarchy, predicts how much ‘social power’ an individual holds and the extent of their leverage [12,13]. Critically, networks are also multi-dimensional, meaning interactions between individuals occur across different contexts including affiliative, agonistic and affinitive behaviours [14]. Given this, and the dynamic nature of networks [5,15], the position of an individual in the social group and their relative contribution to the social structure can vary between networks, and across space and time.

A variety of intrinsic and extrinsic characteristics shape social interactions and, consequently, predict an individual's *current* position in a social network. Amongst others, these include resource distribution [16], predation pressure [17] or demography [18,19]. However, potential *changes* in network structure in response to external factors are often considered opportunistically, owing to the limited flexibility to disrupt social structures in wild populations. For example, following Hurricane Maria, adult rhesus macaques became more affiliative and sought out social connections, with previously more socially isolated monkeys showing the greatest increase in affiliation [20]. In chacma baboons (*Papio hamadryas ursinus*), the death of a high-ranking individual resulted in changes in agonistic interactions but not the grooming network [21], whereas, following a period of large-scale mortality, patterns of affiliation were more resilient in Barbary macaques (*Macaca sylvanus*) than agonistic interactions [22]. Experimental studies, however, allow for highly controlled perturbations that can shed light on a variety of changes in behaviour and its individual- and network-level outcomes.

First, perturbation experiments allow for the identification of keystone individuals in a network. Keystone individuals have a disproportionate impact on overall network structure and functioning (for review, see [10]) and, subsequently, may play more critical roles in processes such as disease transmission [23], the maintenance of social stability [11] and information transmission [24]. Second, controlled manipulations can shed light on the processes underlying social network resilience [25]. For example, Goldenberg *et al*. [26], looked at the response of networks to selective knockouts in African elephants (*Loxodonta africana*). They found that the oldest individuals continued to fill the most socially central positions in the network while daughters replicated the social roles of their mothers. Third, perturbation experiments can illuminate the function and mechanisms of formation of animal social networks. For example, in pig-tailed macaques (*Macaca nemestrina*), knockout experiments showed that policing by a small subset of individuals was critical in maintaining stable networks in the face of chronic perturbations that arise as a result of conflict [27]. Ilany and Akçay [28], theorize the importance of heritability in social network structure by considering the importance of maternal contacts in new-born individual's ultimate network position. Combined, while these studies provide insight into individual-level roles in social networks, they almost exclusively involve the removal of individuals. Few studies consider the result of modifying an individual's behaviour *in situ*, or while still firmly entrenched in their social group.

Differential control over food is very common in social animals and is usually a result of monopolization of general or high-quality food resources by high-ranked individuals (for example, in primates [29–31]). However, in anthropogenic environments in particular, there are other mechanisms that may determine control over food, but this is usually viewed through the lens of social learning, temperament and innovation rather than the social consequences of this control (reviewed in [31]; Griffin *et al*. [32]). For example, certain wild sulphur-crested cockatoos, *Cacatua galerita,* can successfully open garbage bins. At the time of opening, however, multiple cockatoos are present at a single bin thus resulting in the possibility of information transmission in addition to, unintentionally, creating a ‘provider’ individual [33]. Social foraging may also be viewed through the lens of ‘producers’ and ‘scroungers’. Under this framework, producers are individuals who access the food directly, while scroungers obtain food through sharing or aggressive events [34,35]. This has been noted in several species and highlights an important characteristic of social foraging, yet how these relationships modify social interactions is less well understood. There have, however, been experimental studies that have focused more on the social consequences of non-rank-based food control.

Experimental manipulations of a subject's control over food, such as allowing a specific subject novel access to food or a subjects' ability to solve a foraging task, have been used as a means to probe the underlying forces behind non-human primate social interactions and integration while individuals remain in their social group. In vervet monkeys, Fruteau *et al*. [36] modified ‘supply and demand’ by allowing certain individuals the ability to provide food to the group, which resulted in the provider monkeys receiving more grooming than they provided. Kulahci *et al*. [37] investigated how learning influences social network position in ring-tailed lemurs using a novel foraging task. They found that individuals who were frequently observed solving the task received more grooming and thus became more central following the experiment. These studies provide novel insight into the impacts of individual-level, *in situ* perturbations yet stop short of fully exploring the dynamic interplay between individuals, their social capital and social network dynamics.

Fruteau *et al*. [36] and Kulahci *et al*. [37] demonstrated that individuals can flexibly change their short-term social *interactions* with group members in response to increased social leverage (i.e. novel access to food) or to new-found knowledge. However, they focused on changes in one or two behaviours across a relatively short space of time (i.e. days/hours). Given that different behavioural networks, such as grooming, proximity and aggression, have been shown to provide fundamentally different information about social network structure and position [38], and that behavioural networks can also vary widely in their resilience to disturbance [22,39], considering changes in multiple behavioural networks in response to perturbations can provide a more nuanced view of the importance of a given individual in the social network. Also, notably, if the behavioural changes elicited by increased leverage are maintained over a period of time, one might expect changes in network position to be sustained and subsequently cause entire relationships to change.

In this study, we aimed to assess whether allowing an individual to provide high-quality food to the group changed their behavioural relationships by considering changes in (i) individual network position across five, single-layer behavioural networks, (ii) grooming balance, (iii) dominance rank, and (iv) kinship and dominance relationships of the provider and her interaction partners. Combined, these metrics provide a diverse picture of the impact of controlled perturbations on a multi-scale, multi-layer social system.

## 2. Methods

### 2.1. Study site and study subjects

An eight-week study of a resource control experiment was conducted from March to May 2019 on a single social group (formed in 2002) of rhesus macaques (*Macaca mulatta*) housed in a 0.5-acre outdoor corral at the California National Primate Research Center (CNPRC). The group comprised 169 animals (72 females aged 3–24 years old, 11 males aged 3–21 years old, and 86 juveniles and infants), and behavioural observations were focused on all animals 3 years and older. Two animals were permanently removed from the group during the study period (19-year-old female removed during the first week of the experimental phase; 4-year-old male removed during the second week of baseline) and a third individual spent 75% of the 4-week experimental phase out of the group due to health issues (5-year-old female). The corral contained multiple A-frame structures, hanging barrels, and swings. Monkeys were exposed to ambient light and temperature and were fed commercial monkey chow and seed mixture (sunflower seeds, oats) twice daily. All procedures were approved by the UC Davis Institutional Animal Care and Use Committee.

### 2.2. Study design

The study consisted of a four-week baseline period and a four-week experimental period. During the baseline period, behavioural observations were conducted with no modifications to daily routine. During the experimental period, one female subject with average social rank (rank 42 out of 83 adult macaques) and average social connectivity was trained to open a food box and provide food to her group once per day. This female is referred to here as the ‘provider’ monkey. The goal of the experiment was for the monkeys in the group to associate the provider monkey with the availability of the high-quality food resource that was placed inside a feeder box. The provider monkey was trained using standard positive reinforcement techniques, across 12 training sessions from August to December 2018, to pull a non-functional lever on a feeder box with a remotely controlled release operated by research staff.

**Figure 1.**
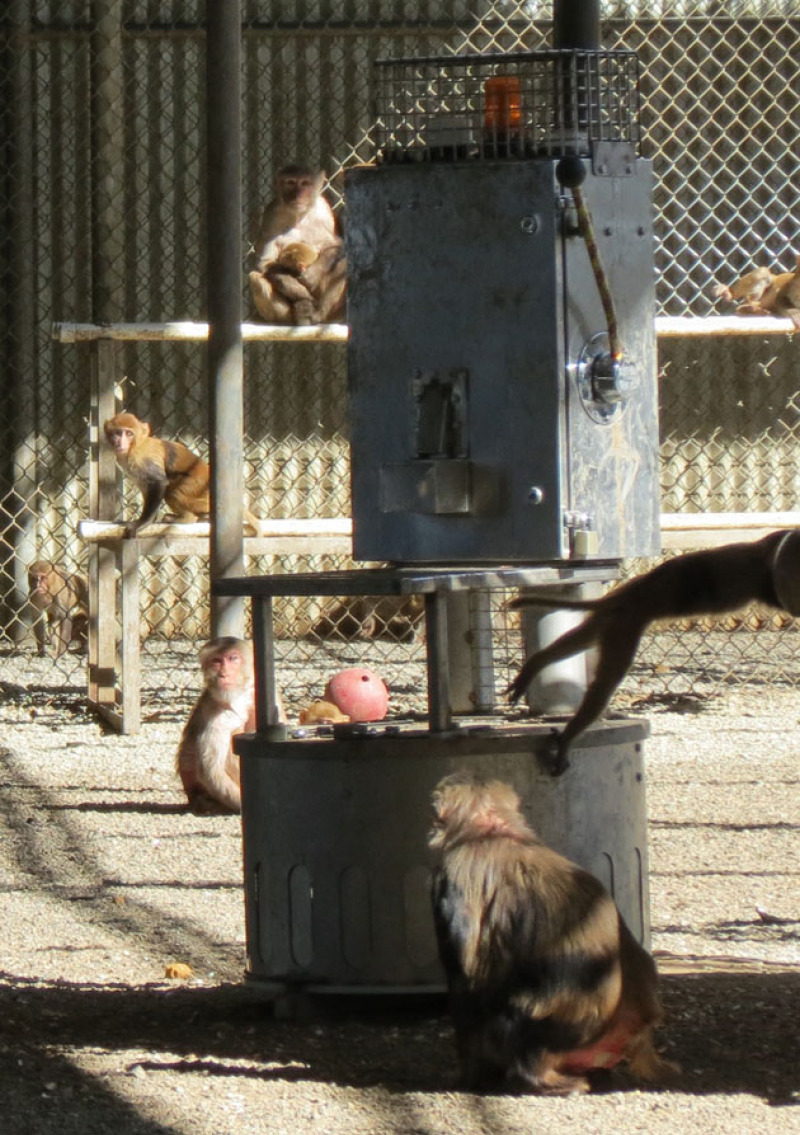
Photo of the feeder box located inside the study group's enclosure.

The feeder box was built in-house by the CNPRC mechanical shop. The primary structure of the box was a cylindrical metal container (approximately 63 cm height × 37 cm diameter) with 16 equally spaced holes around the sides for the monkeys to reach in and grab food items placed inside the cylindrical box (figure 1). The feeder box is opened (and closed) using a rotation mechanism inside the cylinder in which an inner surface of the cylinder rotates approximately 5 cm to open up (or close) the holes. This rotation mechanism is operated via remote control, and the solenoid and wiring were housed in a large rectangular metal box mounted on top of the cylindrical feeder. In addition, a non-functional lever (akin to the pull-down lever of a casino slot machine) was mounted on the right side of the rectangular box, and a strobe light was mounted on top.

The provider monkey was trained to pull the lever when given a verbal and visual cue (i.e. staff saying ‘pull it’ while at the same time turning on the orange strobe light). The observation team, standing outside the enclosure, would wait for the provider monkey to be in proximity to the feeder box and preferably not too close to high-ranking animals that might impede her approach to the feeder box. When the provider monkey pulled the lever on cue, an observer opened the feeder box with the remote control. Because humans are required to put food into the feeder box, there was the potential for the monkeys to associate humans with the food, and not the provider monkey. Therefore, the feeder box was refilled with food the night before the provider monkey opened the feeder box to create a separation between these events.

### 2.3. Behavioural observations

Data were collected for eight continuous weeks, divided into a four-week baseline observation period and a four-week experimental period. The same protocol was followed in both periods. Behavioural observations were conducted by two observers four days per week (Monday–Friday, except Wednesday) on all adult individuals (3+ years) in the group. One observer recorded aggressive interactions (e.g. threats, lunges, and chases) and status signalling interactions (e.g. peaceful approaches that elicited silent bared-teeth displays (SBTs) or move away by the recipient) between adult group members using an ad libitum event sampling design. Aggressive behaviour was categorized as mild (e.g. open mouth threat, short chase less than 6 m), moderate (e.g. grapple/wrestling, chase greater than 6 m), and severe (bite or pin to the ground). Submissive behaviour included freeze/turn away, move away, run away a short distance (less than 6 m), run away a long distance (greater than 6 m), and crouch (recipient is cornered or stops resisting aggression). A second observer recorded all affiliative interactions (i.e. social grooming, huddling/social contact and proximity within arm's reach) between adult group members during scan samples conducted every 20 min. Affiliative interactions were recorded in dyads, and the initiator and recipient specified.

### 2.4. Social network construction and calculation of behaviour variables

A weighted, directed behavioural network was created for each of the following behaviours, for both baseline and experimental phases separately: (i) dyadic aggressive interactions where aggressive behaviour was met with a clear submissive response, (ii) approach–move-away interactions, such as displacements, (iii) SBT signals in response to peaceful approaches (note: peaceful SBTs may be accompanied by other submissive behaviours including turn away, move away or rump present), and (iv) social grooming interactions. Finally, weighted, undirected networks were created for huddling/social contact and proximity interactions. Edge weights for each network were the frequencies of interaction for each dyad. Individual-level measures of centrality were computed for all five networks. For directed behaviours, indegree, outdegree (the number of adjacent edges to each node) and strength (total number of interactions) were calculated, while degree centrality was calculated for undirected networks. There were three animals (two permanent removals; one temporary removal due to health issues) that do not appear at all, or in only a few, of the networks constructed for the experimental phase. These individuals were excluded from group-level means.

### 2.5. Dominance rank

Relative dominance ranks for all adult group members (3 years and older, males and females: *N* = 83) were calculated separately for baseline and experimental phases using the *Perc* package [40] in R to analyse all instances of status signalling (e.g. displacements, SBT in response to approach). Briefly, the *Perc* package uses the network of dominance interactions to fill in the win-loss matrix, and ‘wins’ are calculated using both direct dominance interactions as well as network pathways of interaction (e.g. the pathway of A dominant to B, B dominant to C is used to infer that A is dominant to C).

### 2.6. Statistical analyses

#### 2.6.1. Changes in social network position

To examine whether, and how, the provider monkey's social network position changed in response to the experiment, we compared the provider monkey's baseline network strength and degree centrality (i.e. indegree and outdegree for aggression, displacements, peaceful SBTs and grooming networks; degree for huddling and proximity networks) with her centrality scores during the feeder box experiment. We used the distribution of strength and degree centrality scores for all other group members as guide for interpretation. Further, we calculated the change in network centrality and strength (e.g. experimental minus baseline centrality) for each adult group member in each network, and then compared the provider monkey's magnitude of change with the distribution of scores of all other group members. For this comparison, we calculated *z*-scores by comparing the provider's change (experimental minus baseline) in strength and degree centrality with the mean group change for each network. We then computed two-tailed *p*-values as we made no *a priori* assumptions about the direction of change in the provider's network position and took a conservative approach.

In addition to her position in the grooming network, we compared the provider monkey's grooming balance (grooming received minus grooming given) between the baseline and experimental periods. That is, we looked at the change in proportion of grooming she received and grooming she gave, with a larger balance indicating grooming shifted in her favour.

#### 2.6.2. Change in interaction partners

To determine whether the provider monkey changed *who* she interacted with, in addition to, or instead of, changing the frequency of interaction or number of social partners, we investigated the kinship and dominance relationships of the provider and her interaction partners. First, we calculated the percentage of higher-ranked individuals (ranked higher/ranked lower) who targeted her with aggression for each period to assess whether high-ranked individuals targeted her more frequently once she acquired the ability to provide food to her group. Additionally, we looked at the change in frequency of interactions with kin versus non-kin partners across affiliative, socio-spatial networks. Kin were defined as part of the same matriline or descended from the same common female ancestor.

## 3. Results

### 3.1. Changes in the provider monkey's strength and degree centrality

Compared with other group members, the provider monkey showed average levels of connectedness in most of the behavioural networks across both the baseline (BL) and experimental (Exp) period (electronic supplementary material, figure S1).

**Figure 2.**
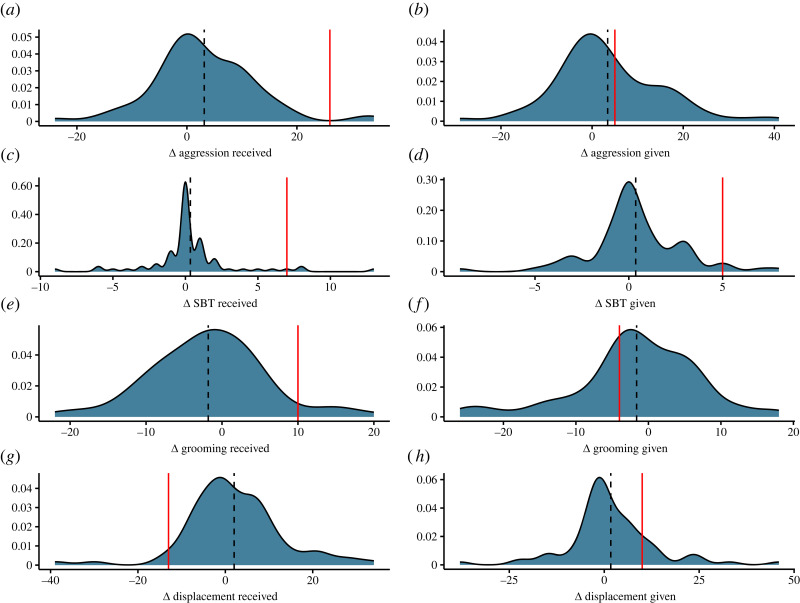
Density plots of the change in node strength (experimental minus baseline) for eight directed behaviours. Vertical red line is drawn to indicate the change in strength observed in the provider monkey and vertical dashed line indicates the mean, group-level change.

The provider monkey showed a threefold increase in the amount of aggression received during the experimental period compared with baseline (strength_BL_ = 10, strength_Exp_ = 36, figure 2*e*). This was coupled with a significant increase in degree centrality (indegree_BL_ = 10, indegree_Exp_ = 26, table 1). However, the provider monkey did not give more aggression (strength_Exp_ = 18, strength_BL_ = 13, figure 2*f*). The provider monkey received more peaceful SBTs (pSBTs) during the experimental period (strength_Exp_ = 7, strength_baseline_ = 0, figure 2*c*) from a more diverse range of individuals (table 1). While the provider monkey also gave more pSBTs during the experimental phase (strength_Exp_ = 6, strength_BL_ = 1, figure 2*d*) and showed an increase in pSBT degree centrality, this was not significantly different from the mean group-level change (table 1 and figure 2*d*).

**Table 1.** Observed change in degree centrality (experimental minus baseline) across all group members (mean and standard deviation) for each behavioural network as well as the provider monkey's change in degree centrality and associated *Z*-score and *p*-value. Statistically significant results are in italics.

network type	network	mean change	s.d.	provider's change	*Z*-score of provider	*p*-value (2-tailed)
Undirected	Huddling	−4.25	5.06	−6	−0.35	0.73
	Proximity	−3.25	6.15	−4	−0.12	0.90
Directed (in)	*Aggression*	*1.30*	*5.28*	*16*	*2.78*	*0.01*
	Displacement	0.86	5.04	−2	−0.57	0.57
	Grooming	−0.23	3.25	−2	−0.54	0.59
	*SBT*	*0.32*	*2.52*	*7*	*2.65*	*0.01*
Directed (out)	Aggression	1.49	6.98	1	−0.07	0.94
	Displacement	0.80	5.75	3	0.38	0.70
	Grooming	−0.20	3.51	−4	−1.08	0.28
	SBT	0.35	2.03	4	1.80	0.07

The provider monkey received more grooming during the experimental period (strength_BL_ = 12, strength_Exp_ = 22, figure 2*a*) from a wider range of individuals in direct contrast to a mean *decrease* in grooming received at the group level (table 1). However, this was not significantly different to the mean group change (table 1). The provider monkey showed limited variation in the strength and degree centrality of grooming given (figure 2*b* and table 1).

**Figure 3.**
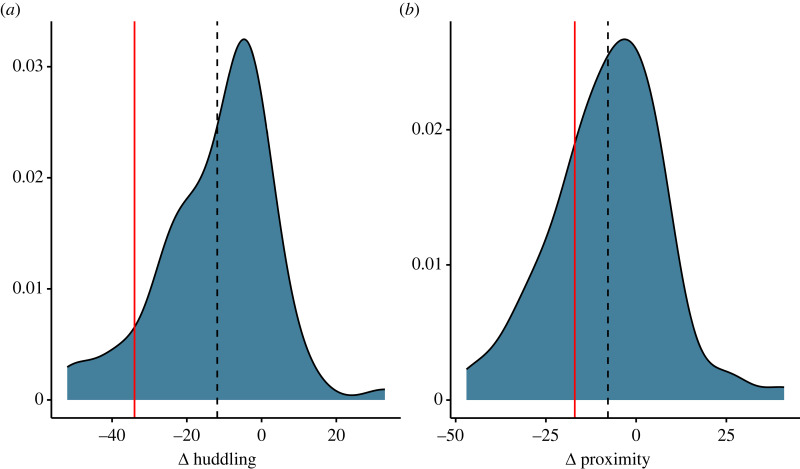
Density plots of the change in node strength (experimental minus baseline) for two undirected behaviours. Vertical red line is drawn to indicate the change strength observed in the provider monkey and vertical dashed line indicates the mean, group-level change.

When considering huddling and proximity, the provider was overall less socio-spatially connected during the experimental period and showed a threefold *decrease* in huddling strength (strength_BL_ = 48, strength_Exp_ = 14, figure 3*a*), accompanied by a decrease in huddling degree centrality (table 1), as well as a decrease in both the frequency of proximity (32 versus 49, figure 3*b*) and degree centrality (table 1). However, these were not significantly different to group mean changes (table 1, figure 3*b*).

At the group-level, behavioural data indicate that during the four-week experimental phase, the average group member showed very little change in degree centrality from baseline to experimental phase with the mean change near zero for all behavioural network measures (table 1).

### 3.2. Changes in the provider monkey's grooming balance

**Figure 4.**
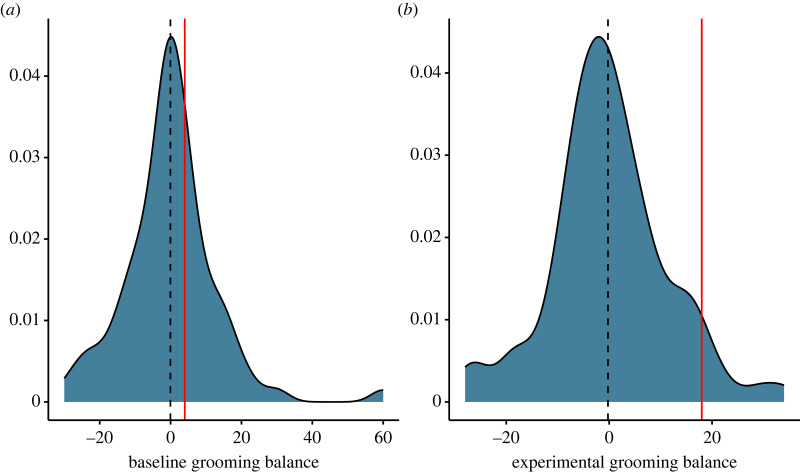
Density plots of grooming balance (grooming received minus grooming given) for baseline (*a*) and feeder box experiment phases (*b*) for all group members, with the provider monkey's value shown by the vertical red line. Vertical dashed line represents the mean group change.

During baseline, most group members showed a grooming balance (grooming received minus grooming given) near zero (mean groom balance = −0.076, s.d. = 12.87). The provider monkey's grooming balance was similar to other animals: she received *slightly* more grooming than she gave (provider's groom balance = 4, *Z*-score = 0.39, figure 4). During the feeder box phase, however, the provider monkey's grooming balance changed compared with other group members, and she received more grooming than she gave (group mean grooming balance = −0.25, s.d. = 11.56, provider's grooming balance = 18, *Z*-score = 1.66. *p* = 0.048). The timing of grooming bouts was independent of the box being opened with only 2 out of 22 bouts of grooming occurring within 60 min of opening the box.

### 3.3. Change in dominance rank and kinship of provider's partners

Examination of the dominance hierarchies for the baseline phase and the experimental phase showed that the group hierarchy showed no significant changes, nor did the provider monkey's dominance rank. The provider monkey was ranked 43rd in both baseline and experimental phases, and the Pearson correlation between animals' baseline ordinal dominance rank and experimental phase dominance rank was *R* = 0.95.

When considering the dominance rank of individuals the provider monkey received aggression from, she showed a 30% increase in aggression directed towards her from higher-ranked individuals compared with a 7.2% mean group change. Further, she showed a minor increase of 4.2% displacements received from higher-ranked individuals versus 0.03% mean group change. The provider monkey also reduced the proportion of aggression that she directed towards higher-ranked individuals from 15.4% during baseline to 5.5% during the experimental period.

Given the provider monkey was overall less socio-spatially connected during the experimental period, we investigated whether this was the result of a decrease in kin or non-kin interactions. After acquiring the ability to provide food to her group, the provider monkey huddled more frequently with non-kin partners than during the baseline period while the proportion of kin versus non-kin partners decreased (table 2). Conversely, the provider monkey spent more time in proximity to kin during the experimental period (43.75% versus 36.73%) and the ratio of kin versus non-kin partners increased slightly from 20.83% during baseline to 25.00% during the experimental period (table 2).

**Table 2.** The count of the provider monkey's total interactions and the percentage of these interactions that involved kin. The count of the provider monkey's unique partners per behaviour network and the percentage of these partners that were kin.

period	behaviour	total interactions	interactions with kin (%)	unique partners	kin partners (%)
BL	huddling	48	52.08	19	21.05
	proximity	49	36.73	24	20.83
Exp	huddling	14	21.43	13	15.38
	proximity	32	43.75	20	25.00

## 4. Discussion

Social network position plays a crucial role in the lives of non-human primates due to the inherent significance of social connections in their evolutionary history and current social dynamics [41–43]. While we have a firmer grasp on the intrinsic and extrinsic factors that shape an individual's *current* network position [16–19], less is known about the possible cause of *changes* in network position. We aimed to experimentally assess whether enabling an individual to provide food to her group would change her social network position and influence who she interacts with. We found that the provider monkey showed marked changes in social network position across multiple networks, both to her potential benefit and likely detriment.

The most conspicuous change was seen in her agonistic interactions which changed across all network and rank measures we considered. She became a more frequent target of aggression from both a wider range of individuals and more high-ranking members of the group. The reasons for this may be twofold. First, this increase in aggression may serve as a means to reinforce the existing dominance hierarchy in the face of a perceived threat, namely, an increase in the provider monkey's social power. While the term ‘social power’ lacks an explicit definition in primate literature (reviewed in [12]), we posit here that in providing food to her group, she gained control of a high-quality food resource which subsequently increased her leverage [13]. This gain would be in direct opposition to the established dominance hierarchy, thus eliciting more frequent aggression from higher-ranked individuals in an attempt to re-establish the status quo.

Alternatively, given the provider monkey was middle-ranked, it is possible that the increase in aggression centrality simply stems from spatial resource defence given she was proximate to the desirable food resource when she opened the feeder box. As with many non-human primate species, aggression is a commonly used strategy by rhesus macaques to defend high-quality resources [44], and the introduction of concentrated food may have increased resource competition. While these contrasting explanations are challenging to tease apart, and are not mutually exclusive, changes in her SBT network position lends credence to the former explanation.

Subordination signals given and received are closely tied to agonistic interactions and we showed changes in subordination signals received and, to a lesser extent, given by the provider monkey. Subordination signals communicate lower-ranked animals' acceptance of their lower social status, yet they use these signals sparingly and only give them to more powerful and well-respected members of the group [11,45,46]. Thus, after gaining control of a highly desired resource, the provider monkey interacted more with higher-ranked animals and was perceived by lower-ranked group members as having greater social power than before gaining control. This points to a possible group-level shift in power dynamics which, although not within the scope of this study, does indicate a key future investigative avenue to understand network-level changes in response to perturbations.

The provider monkey also received social benefits during the experimental phase. While her overall position did not change in the grooming networks considered, she showed a significant change in her grooming balance. That is, her grooming balance shifted to her advantage and she received more grooming than she gave once she had the ability to provide food to her group. In our case, grooming balance shifted as a result of both an increase in grooming received combined with a decrease in grooming given. This may be suggestive of an increase in social capital [47,48] once she had control of a high-quality resource; however, longer-term data would be needed to assess whether any new bonds are maintained. Our findings are largely in agreement with those of Fruteau *et al*. [36] who found that, in the 60 min following the opening of a food container, provider vervet monkeys showed a shift in grooming balance in their favour. Similarly, on the dyadic-level, Stammbach [49] found that long-tailed macaques who provided food to their group received more grooming than they gave to certain individuals. While Fruteau *et al*. [36] propose that the shift in grooming balance may represent a bargaining process, the provider monkeys in their study could open the food container at will, in contrast to the cue provided in our study. Given she had limited control of *when* she opened the box, grooming is unlikely to be used by other individuals to boost access.

Contrary to Fruteau *et al*. [36] and Kulahci *et al*. [37], we found no evidence of a short-term change in the frequency of grooming immediately after opening the box, suggesting that providing food to her group did not result in an immediate social gain. This is probably due to our provider's limited control over *when* she opened the feeder box. That is, grooming does not form part of a bargaining process and may rather be indicative of the larger shift in perceived social power and/or social bonds. If her perceived social power increased, other individuals would be well-served to improve their affiliative bond with her, and those with existing bonds would probably strengthen them. This suggests an adaptive change in long-term dyadic bonds and/or new ‘friendships’ [50]; however, examining this would require a longer-term study. Nevertheless, our results point to a broader underlying cause of changes in affiliative behaviour not fully captured in past studies.

Lastly, while less clear, the dynamics of the provider monkey's other socio-spatial interactions, in the form of huddling and proximity, changed. For both the huddling and proximity networks, strength and degree centrality decreased; however, she spent more time in proximity to kin. This is perhaps not surprising given the stark increase in aggression received and the resultant risk to others of being proximate to direct aggression and potential redirected aggression. Redirected aggression is prevalent in rhesus macaques [51], and increased aggression has deleterious effects [44,52]. Subsequently, while kin bonds are essential in rhesus macaques [53], other individuals may forgo extending periods of spatial proximity to the provider in favour of less risky short-term grooming bouts.

It should be noted that, while we found evidence that the provider's socio-spatial associations changed during the experimental period, we only considered the changes in a single provider. It is possible that the changes seen may be a result of individual-level changes in response to other intrinsic or extrinsic factors not considered. This highlights the need to repeat this study on a larger number of provider monkeys to further validate our results. We anticipate that this could be achieved in future replications of this study.

These findings provide an additional possible lens through which to view control over food in wild populations. While preferential access to food through learning novel foraging techniques in anthropogenic environments might be beneficial to the ‘provider’, an understanding of the knock-on social consequences is lacking.

Taken together, our results offer a preliminary investigation into how *in situ*, experimental manipulations can modify social network position in a social primate. Particularly, how these changes are not uniform across different social networks and point to at least one potential network-level change. This provides the basis for further examination of how these individual-level changes may elicit broader network-level changes and whether these changes are maintained across time.

## Data Availability

Data generated or analysed during this study are available in the Dryad repository: https://doi.org/10.5061/dryad.tb2rbp07s [54]. R notebook is available in the electronic supplementary material [55].
